# 5-(2,6-Dimethoxy­phen­oxy)-2-methyl­sulfanylmethyl-2*H*-tetra­zole

**DOI:** 10.1107/S1600536809006138

**Published:** 2009-02-28

**Authors:** Nader Noroozi Pesyan, Negar Omidkhah, Mina Maghsoodi, Brian O Patrick

**Affiliations:** a Department of Chemistry, Faculty of Science, Urmia University, 57159 Urmia, Iran; b Department of Chemistry, University of British Columbia, Vancouver, BC, Canada V6T 1Z1

## Abstract

In the title mol­ecule, C_11_H_14_N_4_O_3_S, the tetra­zole and benzene rings are nearly perpendicular to each other, forming a dihedral angle of 104.93 (14)°. The crystal packing exhibits weak inter­molecular C—H⋯O hydrogen bonds.

## Related literature

For a related crystal structure, see: Dabbagh *et al.* (2005 ▶).
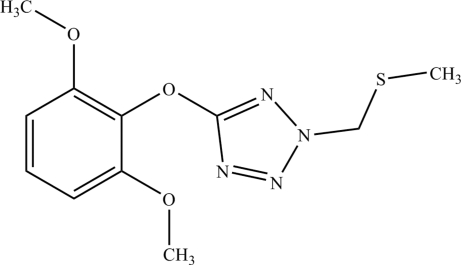


## Experimental

### 

#### Crystal data

C_11_H_14_N_4_O_3_S
*M* *_r_* = 282.32
Orthorhombic, 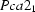

*a* = 12.1795 (5) Å
*b* = 11.0809 (4) Å
*c* = 9.9026 (4) Å
*V* = 1336.45 (9) Å^3^
*Z* = 4
Mo *K*α radiation
μ = 0.25 mm^−1^
*T* = 173 K
0.50 × 0.25 × 0.10 mm

#### Data collection

Bruker X8 APEXII diffractometer
Absorption correction: multi-scan (*SADABS*; Bruker, 2008 ▶) *T*_min_ = 0.871, *T*_max_ = 0.975
7950 measured reflections
2873 independent reflections
2670 reflections with *I* > 2σ(*I*)
*R*_int_ = 0.020

#### Refinement

*R*[*F* ^2^ > 2σ(*F* ^2^)] = 0.026
*wR*(*F* ^2^) = 0.067
*S* = 1.08
2873 reflections
175 parameters
1 restraint
H-atom parameters constrained
Δρ_max_ = 0.18 e Å^−3^
Δρ_min_ = −0.15 e Å^−3^
Absolute structure: Flack (1983 ▶), 1220 Friedel pairs
Flack parameter: 0.06 (6)

### 

Data collection: *APEX2* (Bruker, 2008 ▶); cell refinement: *APEX2*; data reduction: *SAINT* (Bruker, 2008 ▶); program(s) used to solve structure: *SIR* (Altomare *et al.*, 1999 ▶); program(s) used to refine structure: *SHELXTL* (Sheldrick, 2008 ▶); molecular graphics: *ORTEP-3* (Farrugia, 1997 ▶); software used to prepare material for publication: *SHELXTL*.

## Supplementary Material

Crystal structure: contains datablocks I, global. DOI: 10.1107/S1600536809006138/cv2515sup1.cif

Structure factors: contains datablocks I. DOI: 10.1107/S1600536809006138/cv2515Isup2.hkl

Additional supplementary materials:  crystallographic information; 3D view; checkCIF report

## Figures and Tables

**Table 1:** Hydrogen-bond geometry (Å, °)

*D*—H⋯*A*	*D*—H	H⋯*A*	*D*⋯*A*	*D*—H⋯*A*
C10—H10*B*⋯O1^i^	0.99	2.45	3.4277 (19)	170
C11—H11*C*⋯O3^i^	0.98	2.45	3.421 (2)	172
C11—H11*B*⋯O1^ii^	0.98	2.52	3.471 (2)	163
C10—H10*A*⋯O2^iii^	0.99	2.52	3.312 (2)	137

Symmetry codes: (i) 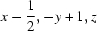; (ii) 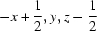; (iii) 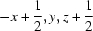.

**Fig. 1.**
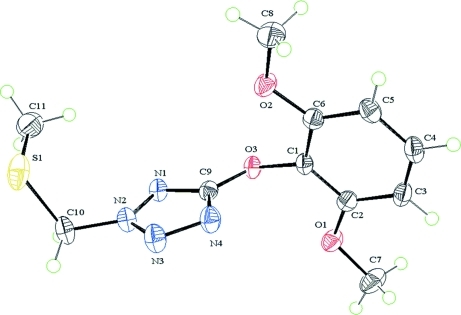
The molecular structure of the title compound showing the atomic numbering and 50% probability displacement ellipsoids.

## supplementary crystallographic information

### Comment

In continuation of our structural study of tetrazole derivatives (Dabbagh *et al.*, 2005) we report herein the structure of the title compound, (I).

In (I) (Fig. 1), the methylsulfanylmethylation proceeded at N2 atom on tetrazole ring. Because of the conjugation of O3 with tetrazole ring the bond distance O3—C9 [1.3388 (17) Å] is obviously shorter than O3—C1 [1.4062 (15) Å]. A similar effect has been found in 5-(4-nitrophenoxy)-1-methylsulfanylmethyl-1*H*-tetrazole (Dabbagh *et al.*, 2005). Tetrazole ring in (I) is planar, and 2,6-dimethoxyphenoxy group deviates from the tetrazole ring plane so torsion angles C6—C1—O3—C9 and C1—O3—C9—N4 are -79.52 (17)° and -9.6 (2)°, respectively. The torsion angle O3—C1—C2—C3 of 174.02 (13)° implies steric interaction between the benzene and tetrazole rings. The S1—C10 bond [1.792 (2) Å] is slightly shorter than C11—S1 [1.798 (2) Å] in methylsulfanylmethyl group.

The crystal packing exhibits weak intermolecular C—H···O hydrogen bonds (Table 1).

### Experimental

Dry DMSO, 5 ml, was added dropwise over a period of 30 min to a solution of 0.143 g of compound in 4 ml of acetic anhydride. The mixture was stirred for 40 h at 45–50°C, excess DMSO and acetic anhydride were removed under reduced pressure, and the residue was washed with several 2–3-ml portions of water. The precipitate was dissolved in 20 ml of methylene chloride, the solution was dried over calcium chloride and evaporated, and the residue was separated by column chromatography on silica gel to isolate compound. Yield 20%. IR spectrum (KBr), ν, cm^-1^: 3050, 2975, 1590, 1530, 1390, 1370, 1300, 1260, 1180, 1110, 760. ^1^H NMR (300 MHz, DMSO-*d*_6_), δ: 7.14 (t, 1H, J = 10 Hz), 6.65 (d, 2H, J = 10 Hz), 5.48 (s, 2H), 3.71 (s, 6H), 2.17 (s, 3H). 13 C NMR (75 MHz, DMSO-d6), δ: 177.50, 152.29, 131.80, 126.74, 105.44, 78.88, 56.20, 56.03, 15.48. Mass spectrum (EI), *m*/*z* (Irel, %) 284 (0.6) [*M* + 2]^+^, 282 (15) [*M*]^+^, 281 (20), 236 (83), 151 (73), 140 (39), 107 (59), 43 (100).

### Refinement

All H atoms were geometrically positioned (C—H 0.95–0.99 Å), and allowed to ride on their parent atoms, with *U*_iso_(H) = 1.2–1.5 *U*_eq_(C).

### Crystal data

**Table:**

C_11_H_14_N_4_O_3_S	*F*(000) = 592
*M**_r_* = 282.32	*D*_x_ = 1.403 Mg m^−^^3^
Orthorhombic, *P**c**a*2_1_	Mo *K*α radiation, λ = 0.71073 Å
Hall symbol: P 2c -2ac	Cell parameters from 4562 reflections
*a* = 12.1795 (5) Å	θ = 2.8–27.8°
*b* = 11.0809 (4) Å	µ = 0.25 mm^−^^1^
*c* = 9.9026 (4) Å	*T* = 173 K
*V* = 1336.45 (9) Å^3^	Irregular, colourless
*Z* = 4	0.50 × 0.25 × 0.10 mm

### Data collection

**Table:**

Bruker X8 APEXII diffractometer	2873 independent reflections
Radiation source: fine-focus sealed tube	2670 reflections with *I* > 2σ(*I*)
graphite	*R*_int_ = 0.020
Area–detector scans	θ_max_ = 28.0°, θ_min_ = 1.8°
Absorption correction: multi-scan (*SADABS*; Bruker, 2008)	*h* = −16→15
*T*_min_ = 0.871, *T*_max_ = 0.975	*k* = −14→14
7950 measured reflections	*l* = −12→12

### Refinement

**Table:**

Refinement on *F*^2^	Secondary atom site location: difference Fourier map
Least-squares matrix: full	Hydrogen site location: inferred from neighbouring sites
*R*[*F*^2^ > 2σ(*F*^2^)] = 0.026	H-atom parameters constrained
*wR*(*F*^2^) = 0.067	*w* = 1/[σ^2^(*F*_o_^2^) + (0.0323*P*)^2^ + 0.1529*P*] where *P* = (*F*_o_^2^ + 2*F*_c_^2^)/3
*S* = 1.08	(Δ/σ)_max_ = 0.002
2873 reflections	Δρ_max_ = 0.18 e Å^−^^3^
175 parameters	Δρ_min_ = −0.15 e Å^−^^3^
1 restraint	Absolute structure: Flack (1983), 1220 Friedel pairs
Primary atom site location: structure-invariant direct methods	Flack parameter: 0.06 (6)

### Special details

**Table:**

Geometry. All e.s.d.'s (except the e.s.d. in the dihedral angle between two l.s. planes) are estimated using the full covariance matrix. The cell e.s.d.'s are taken into account individually in the estimation of e.s.d.'s in distances, angles and torsion angles; correlations between e.s.d.'s in cell parameters are only used when they are defined by crystal symmetry. An approximate (isotropic) treatment of cell e.s.d.'s is used for estimating e.s.d.'s involving l.s. planes.
Refinement. Refinement of *F*^2^ against ALL reflections. The weighted *R*-factor *wR* and goodness of fit *S* are based on *F*^2^, conventional *R*-factors *R* are based on *F*, with *F* set to zero for negative *F*^2^. The threshold expression of *F*^2^ > σ(*F*^2^) is used only for calculating *R*-factors(gt) *etc*. and is not relevant to the choice of reflections for refinement. *R*-factors based on *F*^2^ are statistically about twice as large as those based on *F*, and *R*- factors based on ALL data will be even larger.

### Fractional atomic coordinates and isotropic or equivalent isotropic displacement parameters (Å^2^)

**Table:**

	*x*	*y*	*z*	*U*_iso_*/*U*_eq_	
C1	0.56871 (11)	0.77249 (11)	0.88143 (15)	0.0219 (3)	
C2	0.65815 (12)	0.75439 (12)	0.96546 (15)	0.0236 (3)	
C3	0.74485 (12)	0.83700 (14)	0.96021 (18)	0.0282 (3)	
H3	0.8075	0.8264	1.0161	0.034*	
C4	0.73884 (12)	0.93401 (13)	0.87333 (17)	0.0296 (3)	
H4	0.7984	0.9893	0.8700	0.035*	
C5	0.64873 (13)	0.95357 (13)	0.79062 (17)	0.0279 (3)	
H5	0.6460	1.0215	0.7322	0.033*	
C6	0.56210 (12)	0.87112 (12)	0.79505 (15)	0.0244 (3)	
C7	0.74036 (16)	0.64203 (15)	1.1436 (2)	0.0417 (4)	
H7A	0.8086	0.6213	1.0966	0.063*	
H7B	0.7216	0.5776	1.2073	0.063*	
H7C	0.7503	0.7179	1.1930	0.063*	
C8	0.45668 (14)	0.97946 (14)	0.6331 (2)	0.0388 (4)	
H8A	0.4621	1.0539	0.6861	0.058*	
H8B	0.3848	0.9761	0.5887	0.058*	
H8C	0.5148	0.9782	0.5646	0.058*	
C9	0.39202 (11)	0.70979 (11)	0.94053 (14)	0.0213 (3)	
C10	0.12256 (13)	0.65157 (14)	1.0324 (2)	0.0334 (3)	
H10A	0.0972	0.6804	1.1217	0.040*	
H10B	0.1216	0.5622	1.0341	0.040*	
C11	0.05905 (16)	0.59888 (17)	0.77160 (19)	0.0451 (4)	
H11A	0.1360	0.6080	0.7445	0.068*	
H11B	0.0113	0.6157	0.6941	0.068*	
H11C	0.0464	0.5162	0.8031	0.068*	
N1	0.30621 (9)	0.63680 (9)	0.92891 (14)	0.0250 (3)	
N2	0.23531 (10)	0.69247 (10)	1.01082 (14)	0.0266 (3)	
N3	0.27358 (11)	0.79161 (11)	1.06706 (16)	0.0328 (3)	
N4	0.37580 (11)	0.80385 (11)	1.02308 (15)	0.0298 (3)	
O1	0.65369 (8)	0.65558 (9)	1.04724 (12)	0.0297 (2)	
O2	0.46880 (9)	0.87735 (9)	0.72061 (12)	0.0316 (3)	
O3	0.48597 (8)	0.68410 (8)	0.87649 (10)	0.0231 (2)	
S1	0.02856 (3)	0.70315 (4)	0.90562 (6)	0.04717 (14)	

### Atomic displacement parameters (Å^2^)

**Table:**

	*U*^11^	*U*^22^	*U*^33^	*U*^12^	*U*^13^	*U*^23^
C1	0.0180 (6)	0.0215 (6)	0.0261 (8)	−0.0025 (5)	0.0015 (6)	−0.0033 (5)
C2	0.0226 (7)	0.0245 (6)	0.0237 (8)	0.0029 (5)	0.0009 (6)	−0.0048 (5)
C3	0.0182 (7)	0.0354 (7)	0.0310 (9)	−0.0003 (5)	−0.0019 (6)	−0.0091 (6)
C4	0.0239 (7)	0.0294 (7)	0.0354 (9)	−0.0069 (5)	0.0059 (6)	−0.0086 (6)
C5	0.0292 (7)	0.0237 (6)	0.0308 (8)	−0.0041 (5)	0.0051 (6)	−0.0009 (6)
C6	0.0239 (7)	0.0249 (6)	0.0243 (8)	0.0007 (5)	0.0008 (6)	−0.0018 (5)
C7	0.0426 (10)	0.0425 (8)	0.0400 (10)	0.0014 (8)	−0.0177 (9)	0.0046 (8)
C8	0.0417 (9)	0.0323 (8)	0.0425 (10)	−0.0001 (7)	−0.0069 (9)	0.0140 (7)
C9	0.0203 (6)	0.0197 (5)	0.0238 (8)	0.0018 (5)	−0.0028 (6)	0.0026 (5)
C10	0.0221 (7)	0.0346 (7)	0.0436 (9)	−0.0046 (6)	0.0078 (7)	−0.0024 (7)
C11	0.0453 (11)	0.0475 (10)	0.0423 (11)	−0.0133 (8)	−0.0079 (9)	0.0117 (8)
N1	0.0201 (5)	0.0251 (5)	0.0298 (7)	−0.0018 (4)	0.0008 (5)	−0.0012 (5)
N2	0.0209 (6)	0.0257 (6)	0.0332 (8)	−0.0011 (5)	0.0028 (5)	−0.0010 (5)
N3	0.0267 (6)	0.0288 (6)	0.0430 (9)	−0.0028 (5)	0.0053 (6)	−0.0072 (5)
N4	0.0244 (6)	0.0267 (6)	0.0382 (8)	−0.0030 (5)	0.0022 (6)	−0.0059 (5)
O1	0.0279 (6)	0.0299 (5)	0.0312 (6)	0.0007 (4)	−0.0075 (5)	0.0025 (4)
O2	0.0296 (6)	0.0292 (5)	0.0361 (7)	−0.0045 (4)	−0.0102 (5)	0.0098 (4)
O3	0.0187 (5)	0.0204 (4)	0.0302 (6)	−0.0028 (3)	−0.0003 (4)	−0.0023 (4)
S1	0.02395 (18)	0.0424 (2)	0.0752 (4)	0.00636 (16)	−0.0048 (2)	0.0022 (2)

### Geometric parameters (Å, °)

**Table:**

C1—O3	1.4062 (15)	C7—H7B	0.9800
C9—O3	1.3388 (17)	C7—H7C	0.9800
N2—C10	1.4618 (19)	C8—O2	1.4330 (18)
S1—C10	1.792 (2)	C8—H8A	0.9800
C6—O2	1.3562 (18)	C8—H8B	0.9800
C2—O1	1.3630 (17)	C8—H8C	0.9800
C1—C2	1.385 (2)	C9—N1	1.3265 (17)
C1—C6	1.390 (2)	C9—N4	1.3393 (18)
C2—C3	1.399 (2)	C10—H10A	0.9900
C3—C4	1.379 (2)	C10—H10B	0.9900
C3—H3	0.9500	C11—S1	1.798 (2)
C4—C5	1.386 (2)	C11—H11A	0.9800
C4—H4	0.9500	C11—H11B	0.9800
C5—C6	1.3964 (19)	C11—H11C	0.9800
C5—H5	0.9500	N1—N2	1.3357 (17)
C7—O1	1.431 (2)	N2—N3	1.3169 (17)
C7—H7A	0.9800	N3—N4	1.3260 (18)
			
C9—O3—C1	116.61 (10)	O2—C8—H8B	109.5
N2—C10—S1	113.52 (12)	H8A—C8—H8B	109.5
C10—S1—C11	100.37 (9)	O2—C8—H8C	109.5
C2—C1—C6	121.93 (12)	H8A—C8—H8C	109.5
C2—C1—O3	118.92 (12)	H8B—C8—H8C	109.5
C6—C1—O3	118.99 (12)	N1—C9—O3	120.17 (11)
O1—C2—C1	116.22 (12)	N1—C9—N4	114.28 (12)
O1—C2—C3	125.32 (13)	O3—C9—N4	125.50 (12)
C1—C2—C3	118.47 (13)	N2—C10—H10A	108.9
C4—C3—C2	119.57 (14)	S1—C10—H10A	108.9
C4—C3—H3	120.2	N2—C10—H10B	108.9
C2—C3—H3	120.2	S1—C10—H10B	108.9
C3—C4—C5	122.18 (13)	H10A—C10—H10B	107.7
C3—C4—H4	118.9	S1—C11—H11A	109.5
C5—C4—H4	118.9	S1—C11—H11B	109.5
C4—C5—C6	118.51 (14)	H11A—C11—H11B	109.5
C4—C5—H5	120.7	S1—C11—H11C	109.5
C6—C5—H5	120.7	H11A—C11—H11C	109.5
O2—C6—C1	115.02 (12)	H11B—C11—H11C	109.5
O2—C6—C5	125.64 (13)	C9—N1—N2	100.09 (11)
C1—C6—C5	119.33 (14)	N3—N2—N1	114.41 (12)
O1—C7—H7A	109.5	N3—N2—C10	121.96 (13)
O1—C7—H7B	109.5	N1—N2—C10	123.56 (12)
H7A—C7—H7B	109.5	N2—N3—N4	106.18 (12)
O1—C7—H7C	109.5	N3—N4—C9	105.04 (12)
H7A—C7—H7C	109.5	C2—O1—C7	116.81 (12)
H7B—C7—H7C	109.5	C6—O2—C8	117.09 (12)
O2—C8—H8A	109.5		
			
C6—C1—O3—C9	−79.52 (17)	N4—C9—N1—N2	−0.13 (16)
N4—C9—O3—C1	−9.6 (2)	C9—N1—N2—N3	0.54 (16)
C6—C1—C2—O1	178.73 (13)	C9—N1—N2—C10	177.63 (14)
O3—C1—C2—O1	−5.86 (18)	S1—C10—N2—N3	93.31 (17)
C6—C1—C2—C3	−1.4 (2)	S1—C10—N2—N1	−83.56 (15)
O3—C1—C2—C3	174.02 (13)	N1—N2—N3—N4	−0.76 (18)
O1—C2—C3—C4	−179.56 (14)	C10—N2—N3—N4	−177.90 (15)
C1—C2—C3—C4	0.6 (2)	N2—N3—N4—C9	0.60 (17)
C2—C3—C4—C5	0.5 (2)	N1—C9—N4—N3	−0.31 (17)
C3—C4—C5—C6	−0.7 (2)	O3—C9—N4—N3	−177.81 (14)
C2—C1—C6—O2	−179.07 (13)	C1—C2—O1—C7	−174.19 (14)
O3—C1—C6—O2	5.51 (18)	C3—C2—O1—C7	5.9 (2)
C2—C1—C6—C5	1.2 (2)	C1—C6—O2—C8	178.13 (14)
O3—C1—C6—C5	−174.26 (13)	C5—C6—O2—C8	−2.1 (2)
C4—C5—C6—O2	−179.84 (14)	N1—C9—O3—C1	173.03 (12)
C4—C5—C6—C1	−0.1 (2)	C2—C1—O3—C9	104.93 (14)
O3—C9—N1—N2	177.52 (12)	N2—C10—S1—C11	78.85 (13)

### Hydrogen-bond geometry (Å, °)

**Table:**

*D*—H···*A*	*D*—H	H···*A*	*D*···*A*	*D*—H···*A*
C10—H10B···O1^i^	0.99	2.45	3.4277 (19)	170
C11—H11C···O3^i^	0.98	2.45	3.421 (2)	172
C11—H11B···O1^ii^	0.98	2.52	3.471 (2)	163
C10—H10A···O2^iii^	0.99	2.52	3.312 (2)	137

Symmetry codes: (i) *x*−1/2, −*y*+1, *z*; (ii) −*x*+1/2, *y*, *z*−1/2; (iii) −*x*+1/2, *y*, *z*+1/2.
